# The impact of clinical placement site, community clinic versus tertiary hospital, on midwifery students’ clinical learning experience in Sierra Leone: a cohort study

**DOI:** 10.1186/s12909-023-04413-y

**Published:** 2023-06-07

**Authors:** Julie Mann, Meredith B. Brooks, Frederica Kella, Laura Euller, Sara Adelman, Mustapha Sonnie, Brittney van de Water

**Affiliations:** 1 Seed Global Health, 20 Ashburton Place, 6th Floor, Boston, MA 02108 US; 2 Boston University School of Public Health, 801 Massachusetts Avenue, 3rd Floor, Boston, MA 02118 US; 3 Seed Global Health Sierra Leone, 10B Murray Town Road, Congo Cross, Freetown, Sierra Leone; 4 Boston College, Connell School of Nursing, 140 Commonwealth Ave, Chestnut Hill, MA 02467 USA

**Keywords:** Midwifery, Students, Clinical Education, Clinical learning experience, Clinical Placement, Sierra Leone, Preceptors, Nursing

## Abstract

**Background:**

In midwifery education, the clinical learning experience (CLE) is a critical component to gaining competency and should comprise greater than 50% of a student’s education. Many studies have identified positive and negative factors affecting students’ CLE. However, few studies have directly compared the difference in CLE based on placement at a community clinic versus a tertiary hospital.

**Methods:**

The aim of this study was to examine how clinical placement site, clinic or hospital, impacts students’ CLE in Sierra Leone. A once 34-question survey was given to midwifery students attending one of four public midwifery schools in Sierra Leone. Median scores were compared for survey items by placement site using Wilcoxon tests. The relationship between clinical placement and student’s experience were assessed using multilevel logistic regression.

**Results:**

Two-hundred students (hospitals students = 145 (72.5%); clinic students = 55 (27.5%) across Sierra Leone completed surveys. Most students (76%, n = 151) reported satisfaction with their clinical placement. Students placed at clinics were more satisfied with opportunities to practice/develop skills (p = 0.007) and more strongly agreed preceptors treated them with respect (p = 0.001), helped improve their skills (p = 0.001), provided a safe environment to ask questions (p = 0.002), and had stronger teaching/mentorship skills (p = 0.009) than hospital students. Students placed at hospitals had greater satisfaction in exposure to certain clinical opportunities including completing partographs (p < 0.001); perineal suturing (p < 0.001); drug calculations/administration (p < 0.001) and estimation of blood loss (p = 0.004) compared to clinic students. The odds of students spending more than 4 h per day in direct clinical care were 5.841 (95% CI: 2.187–15.602) times higher for clinic students versus hospital students. There was no difference between clinical placement sites in regards to number of births students attended (OR 0.903; 95% CI: 0.399, 2.047) or number of births students managed without a preceptor/clinician present (OR 0.729; 95% CI: 0.285, 1.867).

**Conclusion:**

The clinical placement site, hospital or clinic, impacts midwifery students’ CLE. Clinics offered students significantly greater attributes of a supportive learning environment and access to direct, hands-on opportunities for patient care. These findings may be helpful for schools when using limited resources to improve the quality of midwifery education.

**Supplementary Information:**

The online version contains supplementary material available at 10.1186/s12909-023-04413-y.

## Background

Strengthening midwifery education and the quality of midwifery care is one of the most impactful interventions to address maternal and newborn morbidity and mortality. Midwives, as members of an integrated healthcare system, can deliver approximately 90% of essential Sexual Reproductive Maternal Newborn Adolescent Health (SRMNAH) interventions across the life course [1]. It is estimated that if the international community makes efforts to achieve universal coverage of midwife-delivered care, 65% of maternal and neonatal deaths and stillbirths could be averted [2]. However, there is a drastic global shortage of midwives. Currently, the world needs over 900,000 more midwives, with the greatest shortage in sub-Saharan Africa, where the burden of maternal and neonatal mortality and morbidity is highest [1, 3, 4].

Sierra Leone, a country of 8 million people in sub-Saharan Africa, has one of the highest maternal and neonatal mortality rates in the world. Approximately 1 in 20 women die from pregnancy or childbirth related complications in Sierra Leone [3]. Sierra Leone has fewer than 500 midwives, with over 40% working in urban settings and collectively serving less than 15% of the population [5]. The Sierra Leone Government estimates a need for 3,000 midwives (an additional 2,500) in order to provide minimally adequate maternal health care services [5]. Thus, the Sierra Leone Ministry of Health and Sanitation (MoHS) National Nursing and Midwifery Strategic Plan 2019–2023 outlines the urgent need to increase the skills and quantity of midwives in the country [5].

The efforts to scale-up midwifery education in Sierra Leone is not just an issue of graduating more midwives. Upon graduation, midwives must be competent in the International Confederation of Midwives (ICM) Essential Competencies of Midwifery Practice [6]. Access to hands-on, clinical experiences to develop knowledge, skills and behavior are critical in securing these essential ICM competencies; however, this has repeatedly been identified as a weakness in midwifery education globally. In the World Health Organization’s (WHO) Strengthening the Quality of Midwifery Education to Achieve Universal Healthcare Coverage 2030, authors identified that midwifery schools and educators are more confident with theoretical classroom teaching than clinical teaching [7]. The State of the World’s Midwifery 2021 found similar challenges identifying inadequate “hands-on” clinical experience in appropriate clinical practice sites as a key barrier to midwifery education [1, 8–10]. Finally, in the WHO’s 2021 Global Strategic Directions for Nursing and Midwifery report, they call for “ensuring appropriate pre-service clinical learning opportunities” in order to develop effective competency-based midwifery education programs” [11].

Recognizing the importance of clinical education in midwifery training, the Office of the Vice President of Sierra Leone invited Seed Global Health (Seed), an international non-government organization working to strengthen the healthcare workforce in sub-Saharan Africa, to partner with the Ministry of Health and Sanitation (MoHS) to support their efforts to decrease preventable maternal and neonatal mortality through strengthening midwifery training. A targeted needs assessment of midwifery clinical training was performed in 2021 to identify strengths and gaps in midwifery clinical training at health facilities in Sierra Leone from various stakeholders’ perspectives. The results of the assessment helped guide Seed and its partners to work synergistically to strengthen midwifery students’ competencies in the country [12].

Beyond identifying the general strengths and gaps in midwifery clinical training in Sierra Leone, there is an interest to explore the impact that clinical placement environments have on a student’s clinical learning experience (CLE). There are many factors that can positively and negatively influence midwifery and nursing student’s’ CLE. Current research has investigated factors such as the presence of a supportive learning environment [13–16], the quality of the student-preceptor relationship [13, 17–19], supervision at clinical sites [20–26], opportunities for hands-on experience [13, 22, 27, 28], and the severity of patients’ illness and staff workload at a facility [22, 24, 27, 29]. However, how the type of clinical placement site, either a community clinic or a tertiary hospital setting, influences midwifery students’ CLE has not been well studied. A community clinic, hereon referred to as “clinic”, versus a tertiary hospital, hereon referred to as “hospital”, can offer varied experiences for midwifery students, especially during an intrapartum clinical placement.

In Sierra Leone, clinics and hospitals differ greatly. Regardless of where pregnant women present for care, they receive free health care under the Free Health Care Initiative established in 2010. Clinics in Sierra Leone provide only Basic Emergency Obstetric Newborn Care (BEmONC) services such as routine, uncomplicated antenatal, intrapartum and postpartum care as well as basic newborn care. Medically complicated patients are referred to a hospital. There are approximately 1,170 clinics and they are situated in villages or small towns and serve a population of 5,000–20,000 [30]. In general, community clinics are often in rural areas and tend to have lower patient volume and less severely ill patients [31]. The clinics are predominantly staffed by a cadre of health workers with less formal education (Maternal child health aides, Community health officers, nurses) than tertiary hospitals [32].

Hospitals in Sierra Leone offer Comprehensive Emergency Obstetric Newborn Care (CEmONC) services, which include access to operating theaters and ability to perform cesarean sections and blood transfusions. There are approximately 24 in a country of 8 million people [30] These hospitals tend to be staffed by a cadre of health workers with greater formal education (doctors, midwives, nurses, surgical technicians) than clinics [32]. In general, hospitals are often in urban settings and tend to have higher patient volume, more medically complicated patients and increased number of students rotating through [28, 31].

Therefore, the aim of this study was to identify strengths and gaps in the clinical training of midwifery students in Sierra Leone from the student perspective and examine how clinical placement site, clinic versus hospital, impacts the student’s clinical learning experience.

## Methods

### Setting & population

There are four public midwifery schools in the country. Each school enrolls anywhere from 40 to 140 midwifery students per year. Regardless of the location of the midwifery school, students are divided and sent to clinics and hospitals in all 16 districts in the country for their clinical learning experiences. In midwifery education, the clinical learning experience (CLE) is a critical component to gaining competency. The International Confederation of Midwives’ Global Standards for Midwifery Education recommends a student should spend a minimum of 50% of their education in the clinical setting [33]. Midwifery students across all four schools who had completed at least one clinical learning experience were eligible to participate in this study.

### Measures

The study team developed a 34-question survey to assess midwifery student’s CLE. Questions regarding student’s perception of preceptors were adapted from the validated Midwifery Student Evaluation of Practice (MidStep tool) [34]. Development of questions regarding competencies gained during clinical placement were guided by the ICM Essential Competencies for Midwifery Practice as well as the seven signal functions of Basic Emergency Obstetric Newborn Care (BEmONC) [6, 31].

Midwifery students anonymously completed surveys on tablets between April and May 2021. Surveys were a mixture of fill in the blank, multiple choice, yes/no, and Likert scale questions with responses ranging from: strongly disagree, mildly disagree, neutral, mildly agree, and strongly agree. Some variables were combined to create composite outcomes including: number of births attended (continuous), births managed without preceptor/clinician present (none or any), number of hours per day spent in direct clinical care (continuous), number of hours per day spent with preceptor (continuous), and frequency of preceptor feedback on performance (daily or less than daily).

### Analysis

Descriptive statistics included calculating the frequency and median for student demographics and using a Chi-square test or Fisher’s Exact Test to assess differences between students’ clinical placement sites. Additionally, we used Wilcoxon tests to compare medians among Likert Scale answers for satisfaction with clinical placement questions. We also employed multilevel logistic regression, clustered at the district level, to assess the relationship between clinical placement and student’s experience as described by: number of births attended, births managed without preceptor/clinician present, number of hours per day spent in direct clinical care, number of hours per day spent with preceptor, and frequency of preceptor feedback on performance. Finally, we assessed the experiences of students based on clinical placement by comparing which students reported on a variety of challenges to learning, complications seen most frequently at their clinical placement site, and areas students wanted more experience using Chi-square. All analyses were completed using SAS 9.4. Statistical significance was set at p < 0.05.

### Ethics

Ethics approval was obtained in Sierra Leone (approved on February 11, 2021, by the Sierra Leone Ethics and Scientific Review Committee, Freetown, Sierra Leone) and the United States (approved on February 23 2021 by Massachusetts General Hospital in Boston; protocol number 2021P000087).

## Results

A total of 202 students participated in this study. Two students were excluded because their clinical placement site was not reported. There were 145 (72.5%) students placed at hospitals and 55 (27.5%) students placed at clinics who completed surveys. Most students were over 30 years old (53%) and the majority (96.5%) were female (Table 1). Nearly all (90.5%) students were in their first year of midwifery school. All but two students had some experience as a nurse prior to enrolling in their current two-year Diploma Midwifery program; 62.1% of students had one to five years’ experience as a registered nurse with all others having more than six years’ experience. Most students (90%) had only one clinical placement rotation prior to participating in the survey. Students placed in clinics were significantly older, had more prior clinical placements in their midwifery program, and had more experience as nurses compared to students placed at hospitals.

**Table 1:** Demographics of students based on clinical placement site

	Total students (n = 200)	Students at hospitals (n = 145)	Students at clinics (n = 55)	p-value
Age				
20–25 yrs	18 (9.0)	17 (11.7)	1 (1.8)	< 0.001
25–30 yrs	76 (38.0)	66 (45.5)	10 (18.2)	
Over 30 yrs	106 (53.0)	62 (42.8)	44 (80.0)	
Gender				0.396*
Male	7 (3.5)	4 (2.8)	3 (5.5)	
Female	193 (96.5)	141 (97.2)	52 (94.5)	
Year in School				0.676
1st Year	181 (90.5)	132 (91.0)	49 (89.1)	
2nd Year	19 (9.5)	13 (9.0)	6 (10.9)	
Number of years as a nurse (SRN or SECHN) (n = 198)				
1–5 yrs	123 (62.1)	104 (72.7)	19 (34.6)	< 0.001
6–10 yrs	61 (30.8)	30 (21.0)	31 (56.4)	
>10 yrs	14 (7.1)	9 (6.3)	5 (9.1)	
Number of clinical placements in midwifery program thus far (average, [IQR]) (n = 192)				
1	129 (67.2)	105 (75.0)	24 (46.2)	< 0.001
2	39 (20.3)	18 (12.9)	21 (40.4)	
≥3	24 (12.5)	17 (12.1)	7 (13.5)	
District for clinical placement				
Bo	18 (9.0)	6 (4.1)	12 (21.8)	N/A
Bombali	10 (5.0)	9 (6.2)	1 (1.8)	
Bonthe	5 (2.5)	4 (2.8)	1 (1.8)	
Falaba	3 (1.5)	0 (0)	3 (5.5)	
Kailahun	4 (2.0)	4 (2.8)	0 (0)	
Kambia	3 (1.5)	0 (0)	3 (5.5)	
Karene	3 (1.5)	1 (0.7)	2 (3.6)	
Kenema	7 (3.5)	6 (4.1)	1 (1.8)	
Koinadugu	3 (1.5)	1 (0.7)	2 (3.6)	
Kono	8 (4.0)	2 (1.4)	6 (10.9)	
Moyamba	8 (4.0)	4 (2.8)	4 (7.3)	
Port Loko	4 (2.0)	0 (0)	4 (7.3)	
Pujehun	8 (4.0)	2 (1.4)	6 (10.9)	
Tonkolili	2 (1.0)	1 (0.7)	1 (1.8)	
Western Area Rural	56 (28.0)	50 (34.5)	6 (10.9)	
Western Area Urban	58 (29.0)	55 (37.9)	3 (5.5)	

Chi-square test used unless otherwise noted. * indicates Fisher’s Exact Test

Students were placed across all 16 districts in Sierra Leone, with the districts containing the four Schools of Midwifery (Western Area Urban and Western Area Rural, Bo, and Bombali) more heavily populated with students for midwifery placements.

### Student satisfaction with clinical learning environment

Table 2 shows the frequency of student responses regarding their clinical placement and CLE. Table 3 shows the median response and interquartile range of the same student responses regarding their clinical placement and CLE and compares median response between placements in clinics versus hospitals.

**Table 2:** Frequency of student responses to likert scale questions regarding students’ perceptions of clinical learning experience

	1 (strongly disagree)	2 (mildly disagree)	3 (neutral)	4 (mildly agree)	5 (strongly agree)
I was satisfied with my clinical rotation and the opportunities to practice and develop my clinical skills.	17 (8.5)	10 (5.0)	22 (11.0)	69 (34.5)	82 (41.0)
The classroom modules helped prepare me for the clinical placement.	8 (4.0)	6 (3.0)	11 (5.5)	65 (32.5)	110 (55.0)
The time spent in the simulation lab helped prepare me for the clinical placement.	21 (10.5)	12 (6.0)	15 (7.5)	62 (31.0)	90 (45.0)
I had a strong understanding of the clinical competencies I was required to develop and practice before starting my clinical placement.	9 (4.5)	8 (4.0)	13 (6.5)	63 (31.5)	107 (53.5)
I understood who to reach out to if I had concerns or issues during my clinical placement.	8 (4.0)	5 (2.5)	16 (8.0)	60 (30.0)	111 (55.5)
I felt supported by the School of Midwifery during my clinical placement.	14 (7.0)	6 (3.0)	11 (5.5)	65 (32.5)	104 (52.0)
For each of the skills listed, I had access to adequate learning opportunities during the clinical placement.					
a. Medical history taking (n = 199)	15 (7.5)	6 (3.0)	7 (3.5)	41 (20.6)	130 (65.3)
b. Triaging (n = 191)	31 (16.2)	12 (6.3)	33 (17.3)	44 (23.0)	71 (37.2)
c. Monitoring of fetal and maternal well being (n = 180)	10 (5.6)	11 (6.1)	17 (9.4)	41 (22.8)	101 (56.1)
d. Fetal palpation (n = 194)	14 (7.2)	30 (15.5)	22 (11.3)	34 (17.5)	94 (48.5)
e. Venipuncture/cannulation (n = 195)	22 (11.3)	37 (19.0)	23 (11.8)	36 (18.5)	77 (39.5)
f. Completing partographs (n = 199)	53 (26.6)	15 (7.5)	10 (5.0)	35 (17.6)	86 (43.2)
g. Perineal Suturing (n = 193)	61 (31.6)	25 (13.0)	21 (10.9)	39 (20.2)	47 (24.4)
h. Estimation of Blood Loss (n = 197)	45 (22.8)	9 (4.6)	8 (4.1)	46 (23.4)	89 (45.2)
i. Drug calculation and administration (n = 199)	39 (19.6)	8 (4.0)	3 (1.5)	26 (13.1)	123 (61.8)
j. Assisting in operating theater (n = 191)	80 (41.9)	14 (7.3)	5 (2.6)	31 (16.2)	61 (31.9)
My preceptor understood the academic elements of my degree program.	9 (4.5)	6 (3.0)	20 (10.0)	60 (30.0)	105 (52.5)
My preceptor treated me with respect.	4 (2.0)	2 (1.0)	16 (8.0)	36 (18.0)	142 (71.0)
My preceptor helped me improve my clinical skills.	3 (1.5)	2 (1.0)	8 (4.0)	45 (22.5)	142 (71.0)
My preceptor provided a safe environment to ask questions.	2 (1.0)	3 (1.5)	8 (4.0)	47 (23.5)	140 (70.0)
My preceptor was available if I needed them during the placement.	3 (1.5)	6 (3.0)	5 (2.5)	69 (34.5)	117 (58.5)
My preceptor had strong teaching and mentorship skills.	4 (2.0)	1 (0.5)	8 (4.0)	33 (16.5)	154 (77.0)
My preceptor was an advocate for my learning.	3 (1.5)	4 (2.0)	14 (7.0)	73 (36.5)	106 (53.0)

Wilcoxon test used to compare medians

**Table 3:** Median of student responses to likert scale questions regarding students’ perceptions of clinical learning experience

	Median (IQR) Total	Median (IQR) Hospital	Median (IQR) Clinic	p-value
I was satisfied with my clinical rotation and the opportunities to practice and develop my clinical skills.	4 (4,5)	4 (3,5)	5 (4,5)	0.007
The classroom modules helped prepare me for the clinical placement.	5 (4,5)	4 (4,5)	5 (4,5)	0.002
The time spent in the simulation lab helped prepare me for the clinical placement.	4 (4,5)	4 (4,5)	4 (2,5)	0.997
I had a strong understanding of the clinical competencies I was required to develop and practice before starting my clinical placement.	5 (4,5)	5 (4,5)	5 (4,5)	0.770
I understood who to reach out to if I had concerns or issues during my clinical placement.	5 (4,5)	4 (4,5)	5 (4,5)	0.002
I felt supported by the School of Midwifery during my clinical placement.	5 (4,5)	4 (4,5)	5 (4,5)	0.363
For each of the skills listed, I had access to adequate learning opportunities during the clinical placement.				
a. Medical history taking (n = 199)	5 (4,5)	5 (4,5)	5 (5,5)	0.080
b. Triaging (n = 191)	4 (3,5)	4 (3,5)	4 (2,5)	0.799
c. Monitoring of fetal and maternal well being (n = 180)	5 (4,5)	5 (4,5)	5 (4,5)	0.020
d. Fetal palpation (n = 194)	4 (3,5)	4 (3,5)	5 (4,5)	0.015
e. Venipuncture/cannulation (n = 195)	4 (2,5)	4 (2,5)	4 (3,5)	0.152
f. Completing partographs (n = 199)	4 (1,5)	5 (2,5)	2 (1,4)	< 0.001
g. Perineal Suturing (n = 193)	3 (1,4)	3.5 (2,5)	1 (1,4)	< 0.001
h. Estimation of Blood Loss (n = 197)	4 (2,5)	4 (3,5)	4 (1,5)	0.004
i. Drug calculation and administration (n = 199)	5 (3,5)	5 (4,5)	4 (1,5)	< 0.001
j. Assisting in operating theater (n = 191)	3 (1,5)	4 (1,5)	1 (1,4)	0.009
My preceptor understood the academic elements of my degree program.	5 (4,5)	4 (4,5)	5 (4,5)	0.429
My preceptor treated me with respect.	5 (4,5)	5 (4,5)	5 (5,5)	0.001
My preceptor helped me improve my clinical skills.	5 (4,5)	5 (4,5)	5 (5,5)	0.001
My preceptor provided a safe environment to ask questions.	5 (4,5)	5 (4,5)	5 (5,5)	0.002
My preceptor was available if I needed them during the placement.	5 (4,5)	5 (4,5)	5 (4,5)	0.074
My preceptor had strong teaching and mentorship skills.	5 (5,5)	5 (4,5)	5 (5,5)	0.009
My preceptor was an advocate for my learning.	5 (4,5)	4 (4,5)	5 (4,5)	0.072

Wilcoxon test used to compare medians

Most students (76%, n = 151/200) reported satisfaction with clinical placements overall. Students placed in the clinic setting more strongly agreed that they were satisfied with their placement and opportunities to practice and develop skills than students placed in the hospital setting (clinic median score: 5 (IQR 4,5); hospital median score: 4 (IQR 3,5); p = 0.007). Similarly, most students (88%, n = 175/200) agreed or strongly agreed classroom modules prepared them well for clinical placement; however, there was a higher median score for students placed in clinics (clinic median score: 5 (IQR 4,5); hospital median score: 4 (IQR 4,5); p = 0.002). Most students also knew who to contact if any issues or concerns arose during clinical placement (86%, n = 171/200). However, students in the clinical setting more strongly agreed with this statement than students in the hospital setting (clinic median score: 5 (IQR 4,5); hospital median score: 4 (IQR 4,5); p = 0.002).

Three quarters of students mildly or strongly agreed (76%, n = 152/200) that “the time spent in the simulation lab helped prepare them for the clinical placement”. Additionally, 85% (n = 170/200) mildly or strongly agreed that they had “a strong understanding of the clinical competencies they were required to develop and practice before starting their clinical placement”. Finally, 85% (n = 169/200) of students reported feeling “supported by the School of Midwifery during my clinical placement”. There was no difference between students’ median score to these statements when comparing student clinical placement sites (hospital vs. clinic).

### Access to clinical learning opportunities

Overall, students reported they were satisfied with the adequate learning opportunities during clinical placements. 86% (n = 171/200) of students agreed (mildly or strongly) they had opportunities for medical history taking, 79% (n = 142/200) reported adequate access to monitoring of fetal and maternal well-being, and 75% (n = 149/200) reported adequate training in drug calculation and administration.

There were some specific skills which students reported statistically significant differences in learning opportunities during their placements, based on clinical placement sites. Students at hospitals and clinics had different opportunities for completing partographs: students at hospitals strongly agreed they had adequate learning opportunities for completing partographs (median score: 5 (IQR 2, 5) versus students placed at clinics who mildly disagreed (median score: 2 (IQR 1, 4); p < 0.001). Other notable skills for which students reported different learning opportunities by their clinical site include: Perineal suturing (hospital median score: 3.5 (IQR 2, 5); clinic median score: 1 (1, 4); p < 0.001); drug calculations and administration (hospital median score: 5 (IQR 4,5); clinic median score: 4 (IQR 1, 4); p < 0.001) and estimation of blood loss (hospital median score: 4 (IQR 3,5); clinic median score: 4 (IQR 1, 5); p = 0.004) were also perceived as better learning opportunities in hospital than clinic settings. Conversely, students perceived opportunities for fetal palpation were greater in the clinic setting compared to hospital settings (hospital median score: 4 (IQR 3,5); clinic median score: 5 (IQR 4, 5); p = 0.015).

Few students were neutral in their assessment of clinical learning opportunities with neutral scores ranging from 1.5% (n = 3/200) for drug calculation and administration to 17.3% (n = 33/200) for triaging. However, some students reported negatively about some aspects of their clinical placements. 34% (n = 68/200) of students mildly or strongly disagreed they had adequate access to completing partographs, mostly among students placed in clinics. Similarly, 23% (n = 45/200) students strongly disagreed they had adequate access to estimation of blood loss; however, this was contrasted with 45% (n = 89/200) who strongly agreed and 23% (n = 46/200) who mildly agreed with adequate access to estimation of blood loss. There was also a lot of inconsistency in responses regarding access to adequate learning in assisting in the operating theater with 42% (n = 80/200) students strongly disagreeing,16% (n = 31/200) mildly agreeing, and 32% (n = 61/200) strongly agreeing with adequate access. The IQRs were between 1 and 5 for all students and students placed in hospitals, while the students in clinics had IQR between 1 and 4. Additionally, there were inconsistencies in responses regarding adequate learning opportunities for perineal suturing with 32% (n = 61/200) students strongly disagreeing and 13% (n = 25/200) mildly disagreeing, 20% (n = 39/200) mildly agreeing and 24% (n = 47/200) strongly agreeing with adequate access. The IQRs were between 2 and 5 for students in hospitals, while the students in clinics IQR was between 1 and 4.

### Student perceptions about preceptors

Most students mildly or strongly agreed that preceptors were supportive at their clinical placement sites. 77% (n = 154/200) strongly agreed that their preceptor had strong teaching and mentorship skills. 71% (n = 142/200) of students strongly agreed that preceptors treated them with respect and helped them improve their clinical skills. 70% (n = 140/200) strongly agreed their preceptor provided a safe learning environment to ask questions.

There were some areas that students did not rate preceptors as strongly, with 30% (n = 60/200) mildly agreeing preceptors understood academic elements of degree program, 35% (n = 69/200) mildly agreeing preceptors were available if they needed them during clinical placement, and 37% (n = 73/200) mildly agreeing preceptors advocated for their learning.

When comparing students’ perceptions of preceptors based on clinical placement sites, there were statistically significant differences. Students in the clinic setting more strongly agreed that their preceptor treated them with respect (clinic median score: 5 (IQR 5, 5)) versus students in the hospital setting (hospital median score: 5 (IQR 4,5), p = 0.001). Also, students in the clinic setting more strongly agreed that preceptors helped improved their midwifery skills (clinic median score: 5 (IQR 5, 5); hospital median score: 5 (IQR 4, 5), p = 0.001), provided a safe learning environment to ask questions (clinic median score: 5 (IQR 5, 5); hospital median score: 5 (IQR 4,5), p = 0.002) and had stronger teaching and mentorship skills (clinic median score: 5 (IQR 5, 5); hospital median score: 5 (IQR 4,5), p = 0.009) than students who had clinical placement sites in the hospital setting.

Beyond students’ perceptions of clinical placement opportunities, we examined the relationship between the type of clinical placement site (hospital or clinic) and student’s access to certain clinical learning experiences and preceptor engagement. (Table 4).

**Table 4:** Relationship between clinical placement site (hospital or clinic) and student clinical learning experience

Outcome	OR (95% CI)	p-value
Greater than ten births attended		
Hospital (REF)		
Clinic	0.903 (0.399, 2.047)	0.8066
Any births managed without preceptor/clinician present		
Hospital (REF)		
Clinic	0.729 (0.285, 1.867)	0.5084
Spending at least 4 h per day in direct clinical care		
Hospital (REF)		
Clinic	5.841 (2.187, 15.602)	0.0005
Spending at least 4 h per day spent with preceptor		
Hospital (REF)		
Clinic	2.083 (0.934, 4.646)	0.0729
Receives daily feedback on performance		
Hospital (REF)		
Clinic	1.207 (0.646, 2.257)	0.5531

*Modeled with hospital as reference; clustered by district

Whether a student was placed in a hospital or clinic setting showed little difference on the number of births they attended (OR 0.903; 95% CI: 0.399, 2.047, p = 0.8066) or whether the student received daily feedback on their performance (OR 1.207; 95% CI: 0.646, 2.257, p = 0.5531). Similarly, there was no difference between placement site when looking at the number of births students managed without a preceptor/clinician present (OR 0.729; 95% CI: 0.285, 1.867, p = 0.5084).

Students who were placed in clinic settings spent a statistically significant more amount of time providing direct clinical care to patients than those in hospital settings. The odds of students spending greater than 4 h per day in direct clinical care were 5.841 (95% CI 2.187–15.602, p = 0.0005) times higher for students in the clinic setting than those students in hospital settings. Although not statistically significant, these same students in the clinic setting also had a higher odds of spending at least 4 h per day with a preceptor than those students in hospital settings (OR: 2.083, 95% CI, 0.934–4.646, p = 0.0729).

In Table 5, the main challenges to learning that students identified were poor housing at their placement and transport to their placement site. These challenges were more of an issue for students in clinic placements versus hospital placements. 58% (n = 32/55) of students at clinics said poor housing was a challenge compared to 20% (n = 29/145) at hospitals (p < 0.001). 73% (n = 40/55) of students at clinic placements identified transport challenges compared to only 31%(n = 45/145) at hospitals (p < 0.001).

**Table 5:** Challenges and Learning Opportunities for Students Based on Clinical Placement Site

	Total students	Students at hospital	Students at clinic	p-value
Main challenges to learning				
lack of time with preceptor	1: 49 (24.5) 0: 151 (75.5)	1: 44 (30.3) 0: 101 (69.7)	1: 5 (9.1) 0: 50 (90.9)	0.002
poor teaching by preceptor	1: 18 (9.0) 0: 182 (91.0)	1: 15 (10.3) 0: 130 (89.7)	1: 3 (5.5) 0: 52 (94.6)	0.281
preceptors too busy to teach	1: 31 (15.5) 0: 169 (84.5)	1: 22 (15.2) 0: 123 (84.8)	1: 9 (16.4) 0: 46 (83.6)	0.835
intimidating learning environment (made to feel bad if don’t know something)	1: 20 (10.0) 0: 180 (90.0)	1: 13 (9.0) 0: 132 (91.0)	1: 7 (12.7) 0: 48 (87.3)	0.429
poor housing at placement	1: 61 (30.5) 0: 139 (69.5)	1: 29 (20.0) 0: 116 (80.0)	1: 32 (58.2) 0: 23 (41.8)	< 0.001
poor transport to placement	1: 85 (42.5) 0: 115 (57.5)	1: 45 (31.0) 0: 100 (69.0)	1: 40 (72.7) 0: 15 (27.3)	< 0.001
too many students per preceptor	1: 42 (21.0) 0: 158 (79.0)	1: 36 (24.8) 0: 109 (75.2)	1: 6 (10.9) 0: 49 (89.1)	0.031
lack of hands-on learning	1: 7 (3.5) 0: 193 (96.5)	1: 4 (2.8) 0: 141 (97.2)	1: 3 (5.5) 0: 52 (94.6)	0.396*
lack of support from midwifery school during placement	1: 17 (8.5) 0: 183 (91.5)	1: 5 (3.5) 0: 140 (96.6)	1: 12 (21.8) 0: 43 (78.2)	< 0.001
Other	1: 12 (6.0) 0: 188 (94.0)	1: 6 (4.1) 0: 139 (95.9)	1: 6 (10.9) 0: 49 (89.1)	0.072
Pregnancy or labor complications seen most frequently				
High blood pressure (preeclampsia/eclampsia)	1: 117 (58.5) 4: 83 (41.5)	1: 83 (57.2) 4: 62 (42.8)	1: 34 (61.8) 4: 21 (38.2)	0.558
Hemorrhage	1: 88 (44.0) 4: 112 (56.0)	1: 69 (47.6) 4: 76 (52.4)	1: 19 (34.6) 4: 36 (65.5)	0.097
Infection (sepsis)	1: 94 (47.0) 4: 106 (53.0)	1: 68 (46.9) 4: 77 (53.1)	1: 26 (47.3) 4: 29 (52.7)	0.962
Breech/Fetal malpresentation	1: 78 (39.0) 4: 122 (61.0)	1: 62 (42.8) 4: 83 (57.2)	1: 16 (29.1) 4: 39 (70.9)	0.077
Obstructed Labor	1: 97 (48.5) 4: 103 (51.5)	1: 75 (51.7) 4: 70 (48.3)	1: 22 (40.0) 4: 33 (60.0)	0.139
Have not experienced complicated labor	1: 86 (43.0) 4: 114 (57.0)	1: 66 (45.5) 4: 79 (54.5)	1: 20 (36.4) 4: 35 (63.6)	0.243
Areas you feel you want more experience in				
Prenatal Care	1: 20 (10.0) 0: 180 (90.0)	1: 15 (10.3) 0: 130 (89.7)	1: 5 (9.1) 50 (90.9)	0.792
Standard labor and delivery	1: 141 (70.5) 0: 59 (29.5)	1: 100 (69.0) 0: 45 (31.0)	1: 41 (74.6) 0: 14 (25.5)	0.440
Management of hemorrhage	1: 40 (20.0) 0: 160 (80.0)	1: 16 (11.0) 0: 129 (89.0)	1: 24 (43.6) 0: 31 (56.4)	< 0.001
Management of infection	1: 46 (23.0) 0: 154 (77.0)	1: 229 (20.0) 0: 116 (80.0)	1: 17 (30.9) 0: 38 (69.1)	0.102
Management of hypertension	1: 31 (15.5) 0: 169 (84.5)	1: 19 (13.1) 0: 126 (86.9)	1: 12 (21.8) 0: 43 (78.2)	0.128
Management of fetal malpresentation	1: 39 (19.5) 0: 161 (80.5)	1: 21 (14.5) 0: 124 (85.5)	1: 18 (32.7) 0: 37 (67.3)	0.004
Postnatal care	1: 41 (20.5) 0: 159 (79.5)	1: 24 (16.6) 0: 121 (83.5)	1: 17 (30.9) 0: 38 (69.1)	0.025
Neonatal care	1: 30 (15.0) 0: 170 (85.0)	1: 17 (11.7) 0: 128 (88.3)	1: 13 (23.6) 0: 42 (76.4)	0.035
Other	1: 3 (1.5) 0: 197 (98.5)	1: 3 (2.1) 0: 142 (97.9)	1: 0 (0) 0: 55 (100.0)	0.283

1 = yes; 0 = no; 4 = N/A

Lack of time with preceptors and too many students per preceptor were also significant barriers that inhibited student learning. These challenges were more prevalent for students in hospital placements. 30% (n = 44/145) of students at hospitals reported lack of time with preceptors as a challenge as opposed to only 9% (n = 5/55) of students at clinics (p = 0.002). Additionally, 25% (n = 36/145) of students reported too many students for each preceptor at hospitals as another challenge as opposed to 11% (n = 6/55) of students at clinics (p = 0.031). Students placed at clinics also reported a lack of support from midwifery schools as a significant challenge compared to students placed at hospitals (p < 0.001).

There was no difference between exposure to certain pregnancy complications based on clinical placement site. However, there were statistically significant differences in students’ desire for more experience based on clinical placement. Students placed in the clinic setting reported wanting more experience in management of hemorrhage (p < 0.001), fetal malpresentation (p = 0.004), postnatal care (p = 0.025) and neonatal care (p = 0.035) compared to students placed at hospitals. The majority of students (70%, n = 141/200), regardless of clinical placement site, reported that they wanted more experience in standard labor and delivery.

## Discussion

Strengthening midwifery students’ clinical competency and confidence is an essential component of addressing maternal morbidity and mortality. However, to our knowledge, there are no studies to date examining the impact of clinical placement sites on midwifery students’ CLE, specifically in a low resource setting. Our study found that regardless of clinical placement site, most midwifery students reported positive CLE across all 16 districts in Sierra Leone. However, there were some statistically significant differences noted between students placed in clinics versus hospitals. Students placed in clinic settings expressed greater satisfaction with their CLE in terms of opportunities to practice and develop skills. They also reported spending significantly more time in direct clinical care with patients than students placed in hospitals. Students’ odds of spending greater than 4 h a day directly caring for patients was 5 times greater if they were in a clinic than a hospital.

Access to hands-on, direct clinical care is critical when developing clinical competency and confidence. The State of the World’s Midwifery 2021 cited inadequate “hands-on” clinical experience in appropriate clinical practice sites as a key barrier to midwifery education [1, 7–10]. Multiple studies assessing nursing and midwifery students CLE have repeatedly found that lack of opportunity to hands-on, individual learning opportunities negatively impact student’s CLE [13, 22, 27, 28]. Conversely, environments that allow midwifery students to have more opportunities for direct clinical care consistent with midwifery models of continuity of care experiences have been found to be highly beneficial to student learning, and clinical skills development [35].

This study also found that students in clinic placements felt a greater sense of certain characteristics of a supportive learning environment and of a stronger preceptor-student relationship. Students in clinic placements reported more often than students in hospitals that preceptors treated them with respect, helped them improve their skills, provided a safe learning environment for questions, and exhibited stronger teaching and mentorship skills. These are all significant characteristics of a positive supportive learning environment. This is notable because many studies have found that nursing and midwifery students, especially those in low-resource settings, lack such supportive environments [24]. Studies in Ghana and Greece explored the importance of a supportive learning environment and found that individualized supervision by a clinical preceptor offered more opportunity for tailored learning as opposed to “team” supervision; creating a better CLE [21, 25, 26]. Studies in Iran, Ghana, Malawi, and South Africa all found that a lack of such a supportive environment, defined as a lack of support from clinical advisors and poor student-preceptor relationships that failed to foster respect, trust and openness to ask questions, had a significant negative impact on nursing and midwifery students’ learning experience [13, 17–19].

Supervision at clinical placements and the number of students rotating at one clinical placement site also impact CLE. Our study found that students at hospital placements found lack of time with preceptors and too many students were challenges to learning more than those students in clinic placements. Inadequate supervision of students has been shown to negatively impact student CLE [20, 22, 23]. And, when in situations of inadequate supervision, a student’s CLE was found to be further hindered by perceived fear of harming the patient or being blamed or criticized [35]. In addition, high numbers of students on the ward at the same time has also been shown to have a negative impact on student’s CLE [13, 22, 27, 28].

One factor where students in clinic placements expressed more challenges to their learning was with housing and transportation which is often out of the control of individual students or difficult for universities to negotiate. Housing and transportation for students CLE is often a challenge, especially in countries with limited infrastructure and resources, and has been shown to negatively impact students’ clinical learning experiences [36, 37]. As Sierra Leone and other countries are interested in scaling up the number of midwives trained, issues of transportation and housing are important issues to address to ensure that students feel supported at any clinical placement site. Innovative strategies to overcome this barrier, especially in community clinic settings, should be investigated. In Australia, the Department of Health developed a “Rural Clinical Training and Support program” that allocated resources to rural clinics and successfully improved CLEs for students in community clinic settings and subsequently attracted students to practice in these settings upon graduation [38]. South Africa addressed this issue by developing a “home stay” program where students are hosted by a family within the community in which they work [39].

Exposure to a range of clinical situations and diagnoses is significant to one’s CLE and essential to developing clinical competency and confidence in any field. Students placed in hospitals showed some evidence of greater satisfaction in exposure to certain clinical opportunities (partographs, practice perineal suturing, drug calculation/administration and estimation of blood loss). Also, students who were not in the hospital setting reported wanting significantly more experience in management of hemorrhage, fetal malpresentation, postnatal care and neonatal care than students at hospital settings. These clinical opportunities are important, especially competence and confidence in estimating blood loss and management of postpartum hemorrhage because postpartum hemorrhage is one of the leading causes of maternal morbidity and mortality globally.

However, our research did not find any statistically significant difference in the number of births attended and exposure to certain pregnancy complications, including postpartum hemorrhage, between hospitals or clinics. This is surprising in that many midwifery schools place greater numbers of students in hospitals, especially tertiary care hospitals, because there is a higher volume of patients and medical complications and therefore it is assumed there are more opportunities for learning. Our study finds that this might not be the case. In fact, such hospitals or tertiary care centers with high acuity levels and high volumes where staff workload is high, creates a busy and stressful environment and can be demotivating factors leading to negative CLE [27, 28]. Research in Malawi and Iran found that in such environments, where there are often staffing shortages, midwifery and nursing students may be treated as part of the workforce and students are often given patient care duties beyond their level of training and experience [22, 27, 29].

Despite students in clinic placements reporting greater supervision and a more supportive CLE, it is troubling to find that there is no difference in the number of births attended without a preceptor/clinician present at clinic versus hospital students. All students should be closely supervised and supported during their clinical placements, especially at the critical time of birth when many emergency situations can develop such as fetal distress, hemorrhage, shoulder dystocia, resuscitation of the newborn [33]. Given the importance of clinical supervision of students, it is unclear why it is not happening. Some studies suggest that preceptors/clinicians are busy with direct patient care or administrative tasks [40], other reasons point to staff shortages [41], and others suggest preceptors/clinicians are not being well-trained or supported to precept students [42].

Most students surveyed (90%) were in their first year of the midwifery program and had only completed one clinical placement prior to participating in the study. This fact could limit the generalizability of our findings however, the majority of these students had previous nursing experience. Therefore, most had prior experience in the clinical setting to help contextualize survey questions and evaluate their CLE and preceptors. We did not analyze any associations between demographics and responses irrespective of placement site. Therefore, we cannot address whether different age groups or students with a greater number of clinical placements would produce different findings. Sierra Leone has significantly limited human and material resources and incredibly high morbidity and mortality, making the CLE potentially harder than other higher resourced or less burdened systems, again possibly limiting the generalizability of our findings.

Although ICM’s guidelines, BEmONC signal functions and the MidStep tool were used to guide the development of the study survey, the survey was not externally validated [6, 31, 34]. The survey was only administered one time. Perhaps if there were multiple points of administration, such as at the beginning, middle and end of midwifery students’ education, our findings would be different. Finally, although surveys were completed anonymously, we found a noticeable positive skew when analyzing the data. These findings could be indicative of true perceptions but may also have resulted from inadvertent pressure respondents felt to reflect positively to portray their own clinical competence or to not disrupt the healthcare hierarchy.

## Conclusion

Overall, midwifery students had high satisfaction with their clinical learning experience regardless of clinical placement site, However, there exist statistically significant differences between students’ experience when placed in a clinic versus a hospital. Clinic placement offers greater attributes of a supportive learning environment and access to direct hands-on opportunities for patient care. Although students perceived more opportunities for certain clinical learning experiences at hospitals, there was no significant difference in the number of births or certain pregnancy complications students were exposed to in either placement. Finally, students in both placement settings lack consistent supervision while attending births.

These findings identify strengths and weaknesses of both clinic and hospital placements for midwifery students and can offer guidance to midwifery schools and governments, like Sierra Leone, when determining where to focus limited resources in order to strengthen CLE. For example, if clinic placements offer more supportive preceptors, and hands-on learning CLE, it could be cost-effective for schools or governments to invest more in housing and transportation, one of the main challenges to the CLE identified in this study. Given that students in both placement settings lack consistent supervision while at the critical time of birth, it is important midwifery education programs identify and address factors leading to this finding. Possible further research in this area could explore whether increasing the factors that lead to more positive CLE directly lead to greater clinical competence and confidence. Also, since this is the first study comparing whether clinical placement site affects CLE, there is a need for additional research in this area, especially in countries interested in strengthening the quality of midwifery education.

## Electronic supplementary material

Below is the link to the electronic supplementary material.

Supplement: 34-Question Survey for Midwifery Students

## Data Availability

The datasets used and/or analyzed during the current study are available from the corresponding author on reasonable request.

## List of Abbreviations

SRMNAH: Sexual Reproductive Maternal Newborn Adolescent Health SRMNAH
MoHS: Ministry of Health and Sanitation
ICM: International Confederation of Midwives
WHO: World Health Organization
Seed: Seed Global Health
CLE: Clinical learning experience
BEmONC: Basic Emergency Obstetric Newborn Care
CEmONC: Comprehensive Emergency Obstetric Newborn Care
Clinic: Community clinic
Hospital: Tertiary hospital
Placement: Clinical placement
SRN: State registered Nurse
SECHN: State Enrolled Community Health Nurse
MidStep tool: Midwifery Student Evaluation of Practice tool
