# Supplementary material for: The impact of clinical placement site, community clinic versus tertiary hospital, on midwifery students’ clinical learning experience in Sierra Leone: a cohort study
Source: BMC Med Educ. 2023 Jun 7;23:416. doi: 10.1186/s12909-023-04413-y (PMC10245409; doi:10.1186/s12909-023-04413-y)
Supplement: Supplementary file 1 — Supplement: 34-Question Survey for Midwifery Students [file 12909_2023_4413_MOESM1_ESM.docx]

**Supplement: 34-Question Survey for Midwifery Students**

| **Questions for Students - Survey** | | |
| --- | --- | --- |
| **Survey Questions** | | |
| **Topic** | **Question** | **Response options** |
| **Demographic/general** |  |  |
| 1 | What school do you attend? | Select: Freetown, Bo, Makeni, COMAHS |
| 2 | What year are you in your program? | Select: 1st year, 2nd year, 3rd year |
| 3 | When did you finish your SRN or SECHN qualification? | Year: |
| 4 | How many clinical placements have you had in your training so far? | Select: 1, 2, 3, 4, >4 |
| **The following questions apply to your most recent clinical placement:** | |  |
| 5 | Which district was your placement in? | Select: Bo, Bombali, Bonthe, Kailahun, Kambia, Kenema, Koinadugu, Kono, Moyamba, Port Loko, Pujehun, Tonkolili, Western Area Rural, Western Area Urban |
| 6 | What type of facility was your placement at? | Select: Regional hospital, district hospital, CHC, CHP, private |
| 7 | Which module is your most recent clinical placement connected to? | Select from list of classroom modules: Fundamentals of Midwifery, Professional ethical and legal aspects of midwifery practice, Applied Anatomy and Physiology of Obstetrics, Information Communication and Technology (ICT), Infection Prevention and Control (IPC), Women’s Health, Introduction to Applied Anatomy and Physiology, Pregnancy and Antenatal, Labour and Delivery, Puerperium, Neonatal and Child Care, Emergency Obstetrics and Newborn Care, Postnatal Care, Community Midwifery, Leadership and Management in Midwifery practice, Other: |
| 8 | I was satisfied with my clinical rotation and the opportunities to practice and develop my clinical skills. | Select: (1) Strongly disagree (2) Mildly disagree (3) Neutral (4) Mildly agree (5) Strongly agree |
| 9 | The classroom modules helped prepare me for the clinical placement. | Select: (1) Strongly disagree (2) Mildly disagree (3) Neutral (4) Mildly agree (5) Strongly agree |
| 10 | The time spent in the simulation lab helped prepare me for the clinical placement. | Select: (1) Strongly disagree (2) Mildly disagree (3) Neutral (4) Mildly agree (5) Strongly agree |
| 11 | I had a strong understanding of the clinical competencies I was required to develop and practice before starting my clinical placement. | Select: (5) Strongly agree (4) Mildly agree (3) Neutral (2) Mildly disagree (1) Strongly disagree |
| 12 | I understood who to reach out to if I had concerns or issues during my clinical placement. | Select: (5) Strongly agree (4) Mildly agree (3) Neutral (2) Mildly disagree (1) Strongly disagree |
| 13 | I felt supported by the School of Midwifery during my clinical placement. | Select: (5) Strongly agree (4) Mildly agree (3) Neutral (2) Mildly disagree (1) Strongly disagree |
| 14 | I had access to adequate learning opportunities during the clinical placement. | Select: (5) Strongly agree (4) Mildly agree (3) Neutral (2) Mildly disagree (1) Strongly disagree |
| 15 | How often did educators or tutors from the school visit the placement? | Select: Never, Once per rotation, 1-2x per rotation, >2x per rotation |
| 16 | On average, how many hours per day did you spend engaged in patient care at your clinical site? | Select: less than 1, 1-2, 2-4, 4-6, 6-8, 8-10, >10. |
| **The following questions are about the mentorship and preceptorship you received during your last clinical placement.** |  |  |
| 17 | Did you have one specific preceptor that worked with you during your clinical rotation? | Select: Y/N |
| 18 | My preceptor understood the academic elements of my degree program. | Select: (1) Strongly disagree (2) Mildly disagree (3) Neutral (4) Mildly agree (5) Strongly agree |
| 19 | My preceptor treated me with respect. | Select: (1) Strongly disagree (2) Mildly disagree (3) Neutral (4) Mildly agree (5) Strongly agree |
| 20 | My preceptor helped me improve my clinical skills. | Select: (1) Strongly disagree (2) Mildly disagree (3) Neutral (4) Mildly agree (5) Strongly agree |
| 21 | My preceptor provided a safe environment to ask questions. | Select: (1) Strongly disagree (2) Mildly disagree (3) Neutral (4) Mildly agree (5) Strongly agree |
| 22 | My preceptor was available if I needed them during the placement. | Select: (5) Strongly agree (4) Mildly agree (3) Neutral (2) Mildly disagree (1) Strongly disagree |
| 23 | My preceptor had strong teaching and mentorship skills. | Select: (5) Strongly agree (4) Mildly agree (3) Neutral (2) Mildly disagree (1) Strongly disagree |
| 24 | My preceptor was an advocate for my learning. | Select: (5) Strongly agree (4) Mildly agree (3) Neutral (2) Mildly disagree (1) Strongly disagree |
| 25 | How often did your preceptor provide feedback on your performance? | Select: Daily, Multiple times per week, Once a week, Once a month, Rarely |
| 26 | How often was that feedback specific and constructive? | Select: (1) Never (2) Rarely (3) Sometimes (4) Very Often (5) Always |
| 27 | On average, how many hours per day did you spend with your preceptor? | Select: <1, 1-2, 2-4, 4-6, 6-8, 8-10, >10 |
| 28 | Other than your preceptor, who provided additional support or mentorship during the placement? | Select all that apply: No one, Another preceptor, A midwife who is not a preceptor, SECHN, MCH Aides, Nursing staff, head of maternity ward, other practitioner |
| 29 | How often were the following teaching or training methods used by your preceptor? | Select for the following: Always, Very Often, Sometimes, Rarely, Never - A. Hands on instruction (e.g. student does delivery, preceptor provides real-time feedback)  - B. Demonstration (e.g. student watches preceptor preform delivery) - C. Verbal Instruction (e.g. preceptors talk to students about how you make diagnoses, do tests, etc)  - D. Observation and modelling (observation by preceptor and student) Other methods: (write in) |
| 30 | What were the main challenges to learning at this clinical placement? | Select all that apply: lack of time with preceptor, poor teaching by preceptor, preceptors too busy to teach, intimidating learning environment (made to feel bad if don't know something), poor housing at placement, poor transport to placement, too many students per preceptor, lack of hands-on learning, lack of support from midwifery school during placement, Other: write in |
| **The following questions are about your experience in labour and delivery during all of your clinical placements:** |  |  |
| 30 | How many births have you attended in your placements thus far? | Select: N/A, 1-5, 5-10, 10-20, >20 |
| 31 | Of those births, how many did you attend/manage without a preceptor or clinician present? | None, Some, Many, All |
| 32 | Which pregnancy or labor complications did you see the most? | Please rank the following in order of frequency, with #1 being most common and #4 being least common.  Select: High blood pressure (preeclampsia/eclampsia), Hemorrhage, Infection (sepsis), Breech/fetal malpresentation N/A: Have not experienced complicated labor  Other: (write in) |
| 33 | Which of the following areas do you feel you want more experience in? | Select all that apply: Prenatal care, standard labor and delivery, management of hemorrhage, management of infection, management of hypertension, management of fetal malpresentation, postnatal care, neonatal care, Other: (write in) |
|  | |  |
| 34 | Do you have further comments or feedback you would like to share about your clinical placement experience? | Open-ended |
